# Yersiniabactin Siderophore of Crohn’s Disease-Associated Adherent-Invasive *Escherichia coli* Is Involved in Autophagy Activation in Host Cells

**DOI:** 10.3390/ijms22073512

**Published:** 2021-03-29

**Authors:** Guillaume Dalmasso, Hang Thi Thu Nguyen, Tiphanie Faïs, Sébastien Massier, Caroline Chevarin, Emilie Vazeille, Nicolas Barnich, Julien Delmas, Richard Bonnet

**Affiliations:** 1 M2iSH (Microbes, Intestin, Inflammation and Susceptibility of the Host), Inserm U1071, INRAE USC 2018, Université Clermont Auvergne, CRNH, 63001 Clermont-Ferrand, France; hang.nguyen@uca.fr (H.T.T.N.); tfais@chu-clermontferrand.fr (T.F.); sebastien.massier@gmail.com (S.M.); caroline.chevarin@uca.fr (C.C.); emilie.vazeille@uca.fr (E.V.); nicolas.barnich@uca.fr (N.B.); jdelmas@chu-clermontferrand.fr (J.D.); 2 Laboratoire de Bactériologie, Centre Hospitalier Universitaire, 63001 Clermont-Ferrand, France; 3 Service d’Hépato-Gastro Entérologie, 3iHP, Centre Hospitalier Universitaire, 63000 Clermont-Ferrand, France; 4 Centre de Référence de la Résistance Aux Antibiotiques, Centre Hospitalier Universitaire, 63000 Clermont-Ferrand, France

**Keywords:** Crohn’s disease, AIEC, autophagy, HIF-1alpha, siderophore

## Abstract

Background: Adherent-invasive *Escherichia coli* (AIEC) have been implicated in the etiology of Crohn’s disease. The AIEC reference strain LF82 possesses a pathogenicity island similar to the high pathogenicity island of *Yersinia* spp., which encodes the yersiniabactin siderophore required for iron uptake and growth of the bacteria in iron-restricted environment. Here, we investigated the role of yersiniabactin during AIEC infection. Methods: Intestinal epithelial T84 cells and CEABAC10 transgenic mice were infected with LF82 or its mutants deficient in yersiniabactin expression. Autophagy was assessed by Western blot analysis for p62 and LC3-II expression. Results: Loss of yersiniabactin decreased the growth of LF82 in competitive conditions, reducing the ability of LF82 to adhere to and invade T84 cells and to colonize the intestinal tract of CEABAC10 mice. However, yersiniabactin deficiency increased LF82 intracellular replication. Mechanistically, a functional yersiniabactin is necessary for LF82-induced expression of HIF-1α, which is implicated in autophagy activation in infected cells. Conclusion: Our study highlights a novel role for yersiniabactin siderophore in AIEC–host interaction. Indeed, yersiniabactin, which is an advantage for AIEC to growth in a competitive environment, could be a disadvantage for the bacteria as it activates autophagy, a key host defense mechanism, leading to bacterial clearance.

## 1. Introduction

Crohn’s disease (CD) is a chronic, inflammatory disorder of the gastrointestinal tract caused by microbial and environmental factors in genetically susceptible hosts [1,2]. It has been observed that the intestinal mucosa of CD patients is abnormally colonized by specific *Escherichia coli* strains [3,4,5,6]. Characterization of these *E. coli* strains has showed that they are able to adhere to and to invade intestinal epithelial cells (IECs), as well as to survive and replicate within macrophages [3]. Thus, these strains have been designated as a new pathovar of *E. coli* and called adherent-invasive *E. coli* (AIEC) [3,7]. Once inside the cell, AIEC can proliferate and induce an inflammatory response [8,9].

Implication of AIEC in the etiology of CD has been then largely studied [10]. It has been revealed that AIEC are well adapted for the high nutrient competition with the commensal microbiota [11]. For example, bile salts activate many degradation pathways that promote the growth of AIEC, and this was not observed for commensal bacteria [11]. AIEC also have the ability to colonize the intestinal epithelium. It has been shown that AIEC express a mucinase and a flagellum allowing them to penetrate through the mucus layer to reach the intestinal epithelium [12,13]. Then, AIEC adhere to IECs via the chitinase chiA by recognizing chitinase 3-like-1 (CHI3L1) [14], and the adhesin FimH of type I pili [15,16] by recognizing CEACAM6, a protein abnormally expressed in the IECs of CD patients [17]. Importantly, AIEC colonize the gut and induce intestinal inflammation in a mouse model expressing human CEACAM6, the CEABAC10 transgenic mice [18].

Autophagy, an evolutionarily conserved process that degrades the cytoplasmic components via the lysosomal pathway, has emerged as a key mechanism of host defense against AIEC [19,20]. Dysfunctional autophagy is recognized as a contributing factor in many chronic inflammatory diseases, including CD [19,20]. Our studies have shown that a functional autophagy is required to restrain the replication of AIEC in host cells and that CD-associated polymorphisms in the autophagy-related genes lead to defective autophagy, thus promoting the intracellular persistence of AIEC and an exacerbated pro-inflammatory response [21,22,23]. While host cells induce a functional autophagy to control AIEC replication, via activating the metabolic stress response pathway EIF2AK4–EIF2A–ATF4 [24,25], AIEC can subvert autophagy by up-regulating the levels of specific autophagy-targeting microRNAs, i.e., miR-130a and miR-30c, in IECs [26]. AIEC also inhibit autophagy by impairing host SUMOylation, a eukaryotic-reversible post-translational modification [27], which consequently increases AIEC intracellular replication and inflammation [26,27]. Recently, we showed that upon AIEC infection, IECs and macrophages secrete an increased amount of exosomes, which are extracellular vesicles of 30 to 100 nm playing a role in cell-to-cell communication [28]. Furthermore, the exosomes secreted by AIEC-infected cells can transfer miR-30c and miR-130a from cell to cell, thus inhibiting autophagy in exosome-receiving cells and consequently favoring AIEC replication [29]. Together, these studies show a crucial role for autophagy in host–AIEC interaction.

One important battle inside the organism is the capture of iron, an essential micronutrient involved in oxygen transport, electron transfer, growth, DNA synthesis and that acts as a co-factor for many enzymes [30,31]. Bacteria have developed siderophores which are iron-specific receptors with high affinity and transport systems [32,33]. *E. coli* could harbor more than one siderophore [32]. Among them, yersiniabactin is of particular interest. Yersiniabactin is encoded by the high-pathogenicity island (HPI), a genomic island indispensable for *Yersinia* pathogenicity [34]. In contrast to the majority of pathogenicity islands described so far, the *Yersinia* HPI is widely distributed among different members of the *Enterobacteriaceae*, such as *E. coli* [34]. The AIEC reference strain LF82 harbors several pathogenic islands (PAI) [35]. Interestingly, one of them, PAI II, is highly similar to the HPI of *Yersinia enterocolitica* (with more than 99% sequence similarity) [35]. In CD, intestinal dysbiosis has been observed, and *Enterobacteriaceae*, which are particularly efficient in trapping iron, are frequently over-represented [36]. Indeed, it has been shown that the siderophores of *Enterobacteriaceae* are critical for their survival in an iron-restricted environment, either in the circulation or in inflammation tissues [37].

Here, we investigated whether yersiniabactin could play another role than that of a siderophore in the complex interaction between the CD-associated AIEC and the host.

## 2. Results

### 2.1. PAI II Is Over-Represented in AIEC Strains

**Figure 1:**
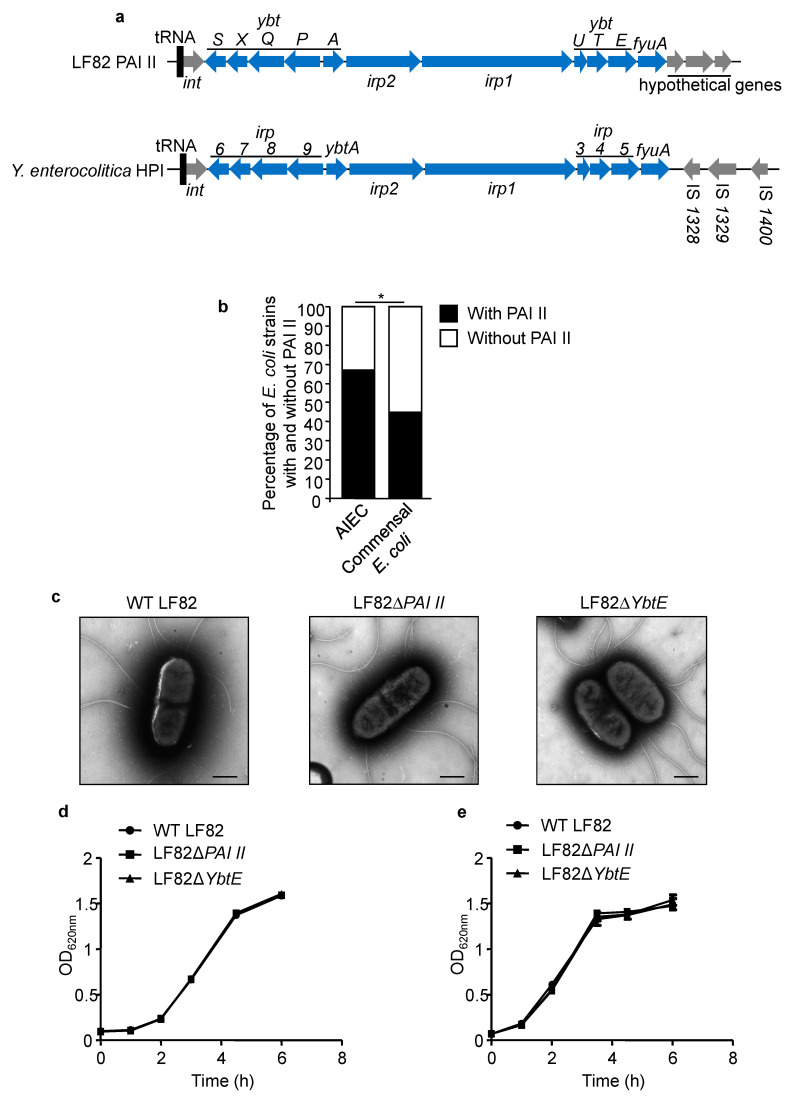
Loss of yersiniabactin does not affect AIEC LF82 morphology and growth in culture media. (**a**) Genetic organization of LF82 pathogenic island (PAI) II showing its similarity to *Yersinia enterocolitica* HPI. The genes at the same position in these two pathogenicity islands share similar sequences but were given different names. (**b**) Number of adherent-invasive *Escherichia coli* (AIEC) or commensal *E. coli* strains with (black bars) and without (white bars) PAI II. (**c**) Transmission electronic microscopy images of negatively stained wild-type (WT) LF82 and mutants LF82Δ*PAI II* and LF82Δ*YbtE*. *Bars* = 500 nm. (**d**,**e**) Bacteria were incubated at 37 °C in LB (**d**) or DMEM/F12 (**e**) medium, and optical density (OD) at 620 nm was measured at the indicated times. Values are means ± SEM of six replicates and are representatives of two independent experiments. * *p* < 0.05; by chi-square test (**b**).

Recently, independent studies have highlighted the genetic variability of CD-associated AIEC strains [38,39,40]. Of interest, the AIEC reference strain LF82 harbors PAI II [35], which shares high sequence similarity with *Y. enterocolitica* HPI (Figure 1a). The core region of LF82 PAI II and *Y. enterocolitica* HPI is composed of 11 genes involved in biosynthesis, regulation, and transport of the yersiniabactin siderophore [34] (shown in blue, Figure 1a). Several genes of LF82 PAI II and *Y. enterocolitica* HPI were given different names but have >99% similarity in sequence (for example, *ybtS* of LF82 PAI II and *irp6* of *Y. enterocolitica* HPI are the same gene). By PCR, we analyzed the presence of PAI II in AIEC strains and in commensal *E. coli* strains isolated from healthy donors. We found a higher prevalence of PAI II in AIEC strains (67.1%, 49/73 strains tested) compared to commensal *E. coli* strains (45%, 18/40 strains tested) (* *p* = 0.022; Figure 1b). This result suggested that PAI II might confer an advantage for AIEC to efficiently colonize the intestinal tract of CD patients.

### 2.2. Loss of Functional Yersiniabactin Does Not Alter AIEC Morphology and Growth

As iron concentration controls numerous signaling pathways [30], we investigated whether PAI II could affect the morphology and growth of AIEC. To test our hypothesis, we constructed a genomic mutant of the AIEC reference strain LF82 that lacks the entire PAI II (LF82Δ*PAI II*) and a mutant lacking *YbtE* (LF82Δ*YbtE*), a gene required for yersiniabactin siderophore production [41]. The loss of the entire PAI II island or of the *YbtE* gene was verified using PAI II and *YbtE* internal primers, respectively (data not shown). Electronic microscopy was used to compare the morphology of wild-type LF82 with those of the mutants LF82Δ*PAI II* and LF82Δ*YbtE*. As shown in Figure 1c, the mutants LF82Δ*PAI II* and LF82Δ*YbtE* did not display any abnormalities in morphology compared with wild-type LF82. Furthermore, loss of PAI II did not alter the growth of LF82 bacteria either in LB or in DMEM/F12 used for IEC culture (Figure 1d,e). These results suggested that loss of PAI II does not affect AIEC LF82 morphology and growth.

### 2.3. Loss of Functional Yersiniabactin Impairs AIEC Growth and Colonization of IECs in Competitive Conditions

**Figure 2:**
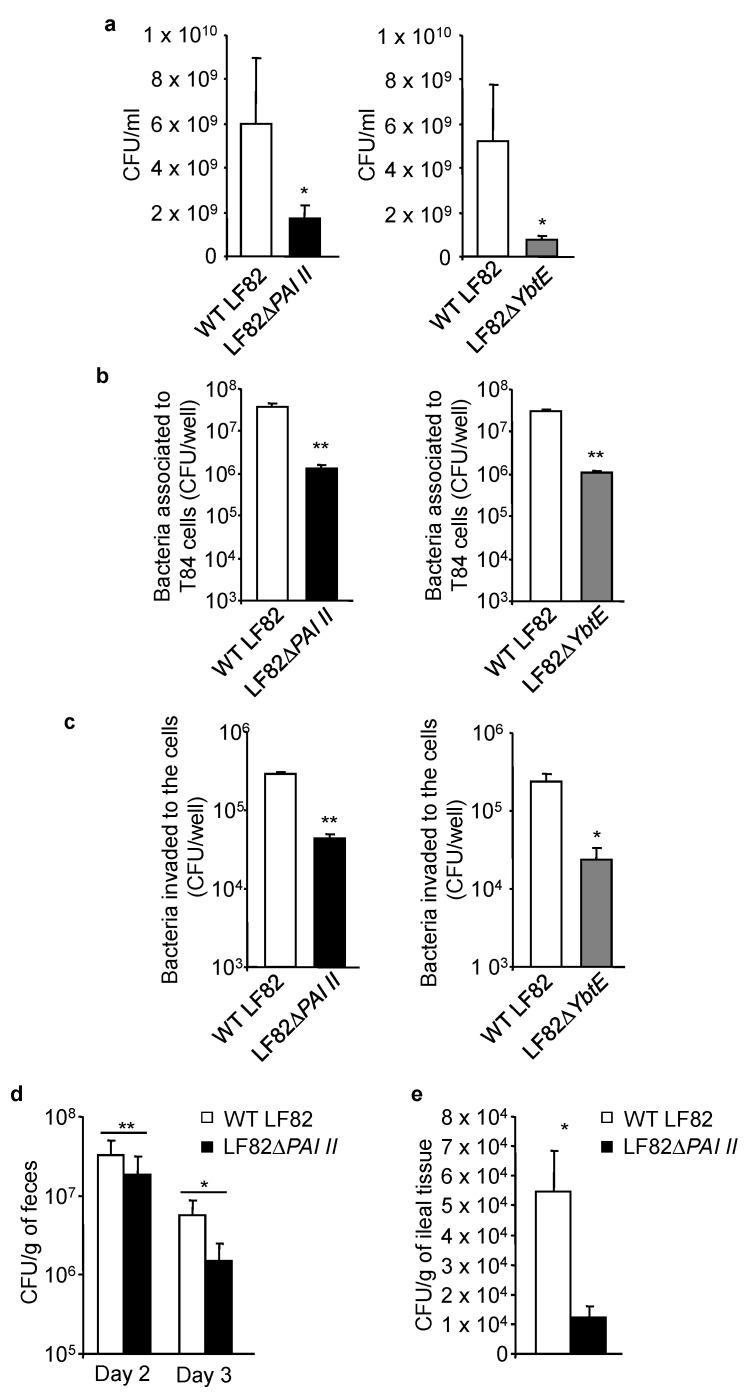
Loss of yersiniabactin affects AIEC LF82 growth in competition experiments. (**a**) The same amount (100 colony-forming units (CFU)) of LF82Δ*PAI II* or LF82Δ*YbtE* mutant was incubated with wild-type (WT) LF82 in 10 mL of LB medium. After one night, bacterial number was counted using LB agar plates. Data are means ± SEM of four replicates and are representatives of three independent experiments. * *p* < 0.05; by Mann–Whitney test. (**b**,**c**) T84 cells were infected with a combination of the same amounts of wild-type LF82 and LF82Δ*PAI II* or LF82Δ*YbtE* (10 wild-type LF82 and 10 mutant bacteria per cell). (**b**) After 3 h of infection, the cells were washed, and the number of bacteria associated with the cells was determined. Data are means ± SEM of six replicates and are representatives of two independent experiments. ** *p* ≤ 0.01 by Mann–Whitney test. (**c**) After 3 h of infection, the cells were washed and incubated with gentamicin for 1 h, and the number of bacteria that invaded to the cells was determined. Data are means ± SEM of six replicates and are representatives of two independent experiments. * *p* < 0.05, ** *p* ≤ 0.01 by Mann–Whitney test. (**d**,**e**) CEABAC10 mice were orally infected with the same number (10^9^ CFU) of LF82Δ*PAI II* and wild-type LF82. Quantification of wild-type LF82 and LF82Δ*PAI II* numbers in the feces collected at days 2 and 3 post-infection (**d**) or associated with the ileal mucosa determined on the day of sacrifice (**e**) (day 3 post-infection). Data are means ± SEM of N = 9 mice/group. * *p* < 0.05, ** *p* ≤ 0.01 by Wilcoxon test.

We next investigated the impact of the loss of the yersiniabactin siderophore of LF82 in competition experiments. As shown in Figure 2a, when the mutant LF82Δ*PAI II* or LF82Δ*YbtE* was mixed with the wild-type LF82 strain, their growth in LB was significantly decreased compared to that of the LF82 strain. Next, human intestinal epithelial T84 cells were infected with a combination of the same amounts of wild-type LF82 and LF82Δ*PAI II* or LF82Δ*YbtE*. After 3 h of infection, T84 cells were washed, and the number of associated bacteria, including adherent and invasive bacteria, was determined. To determine the number of invasive bacteria, after 3 h of infection, the cells were washed and incubated with gentamicin for 1 h to kill the extracellular adherent bacteria. As shown in Figure 2b, there was a decrease in the number of the mutant LF82Δ*PAI II* or LF82Δ*YbtE* associated with T84 cells compared to that of wild-type LF82. Furthermore, the number of the mutant LF82Δ*PAI II* or LF82Δ*YbtE* that invaded the cells was also decreased compared to that of wild-type LF82 (Figure 2c).

To validate these data, CEABAC10 transgenic mice were infected by gavage with a combination of the same amounts of wild-type LF82 and LF82Δ*PAI II*. Bacterial colonization was assessed in the feces on days 2 and 3 post-infection and in the ileal tissues collected on the day of sacrifice (day 3 post-infection). We found higher numbers of wild-type LF82 compared to LF82Δ*PAI II*, both in the feces and in the ileal tissue (Figure 2d,e). These results demonstrated that in a competitive environment, PAI II is necessary for AIEC growth and colonization of IECs.

### 2.4. Loss of Functional Yersiniabactin Increases AIEC Intracellular Proliferation and Decreases Autophagy in IECs

**Figure 3:**
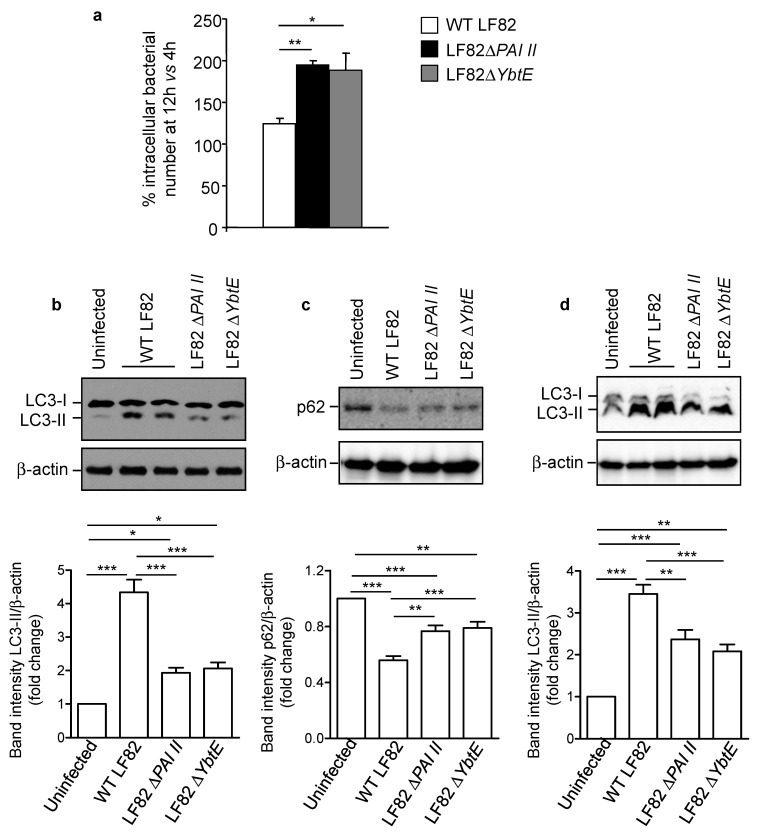
Loss of yersiniabactin decreases autophagy in intestinal epithelial cells (IECs) and increases AIEC LF82 intracellular proliferation. (**a**) T84 cells were infected with either the wild-type (WT) LF82 strain or one of the mutants AIECΔ*PAI II* and AIECΔ*YbtE* for 3 h, washed, and incubated with gentamicin for 1 h or 9 h. The number of intracellular bacteria was counted on LB agar plates and is presented as the percentage of intracellular bacteria at 12 h (3 h infection + 9 h in gentamicin) compared to 4 h (3 h infection + 1 h in gentamicin) post-infection. Data are means ± SEM of six replicates and are representative of two independent experiments. (**b**,**c**) T84 cells were infected with WT LF82, LF82Δ*PAI II*, or LF82Δ*YbtE* strain for 3 h, washed, and incubated with gentamicin for 3 h. Expression of LC3 and p62 was analyzed by Western blot. Bar graphs represent the quantification of bands’ intensity from immunoblots. (**d**) Cells were pre-treated with bafilomycin A1 at 50 nM for 30 min and then infected as in **b** and **c** in cell culture medium containing 50 nM of bafilomycin A1. (**b**–**d**) Data are means ± SEM of three replicates and are representative of three independent experiments. (**a**–**d**) * *p* < 0.05, ** *p* ≤ 0.01, *** *p* ≤ 0.001 by one-way Anova, followed by a post-test Bonferroni.

One of the particularities of AIEC is their ability to survive and to replicate inside host cells [8,9]. We next investigated whether the loss of functional yersiniabactin alters these properties. The number of intracellular LF82 at 12 h versus 4 h post-infection was determined using the gentamicin protection assay and considered as indicative of bacterial intracellular replication. We observed a significant increase in the replication of the mutants LF82Δ*PAI II* and LF82Δ*YbtE* compared to the wild-type LF82 strain in T84 cells (Figure 3a).

As we have previously shown a crucial role for autophagy in the control of AIEC intracellular replication [21,24,25,26,27], we next investigated whether autophagy is implicated in the increase of LF82 intracellular replication associated with the loss of yersiniabactin. Autophagy was assessed by Western blot analysis by evaluating the shift of LC3-I (the cytosolic form) toward LC3-II (the autophagosomal form and a marker of autophagy induction) and the level of p62, an autophagy receptor which is degraded by a functional autophagy [42]. As expected, autophagy was induced in T84 cells upon infection with the wild-type LF82 strain, as evidenced by increased LC3-II level compared with uninfected cells (Figure 3b). Furthermore, induction of a functional and degradative autophagy in LF82-infected cells was confirmed by a decrease in p62 level compared to uninfected cells (Figure 3c). Infection with the mutant LF82Δ*PAI II* or LF82Δ*YbtE* led to increased LC3-II level and decreased p62 level compared to uninfected cells, but with a less pronounced effect compared to infection with the wild-type LF82 strain (Figure 3b,c). These observations suggested a link between the presence of a functional yersiniabactin siderophore and the activation of autophagy in infected cells. The increase in LC3-II levels could result from the activation of autophagy or a blockage of autophagy flux, leading to the accumulation of undegraded LC3-II [42]. Thus, to examine whether the presence of yersiniabactin siderophore led to autophagy activation or autophagy flux blockage, we treated the cells with the inhibitor of autophagy flux bafilomycin A1 before infection. Under autophagy flux inhibition, we still observed the increase in LC3-II level upon infection with the wild-type LF82 strain, and a lower increase in LC3-II level was observed upon infection with the mutant LF82Δ*PAI II* or LF82Δ*YbtE* (Figure 3d). This demonstrated that autophagy was induced in IECs upon LF82 infection and this was dependent, at least in part, on the presence of yersiniabactin siderophore.

### 2.5. Loss of Yersiniabactin Inhibits AIEC-Induced HIF-1α Expression and Activation in IECs

**Figure 4:**
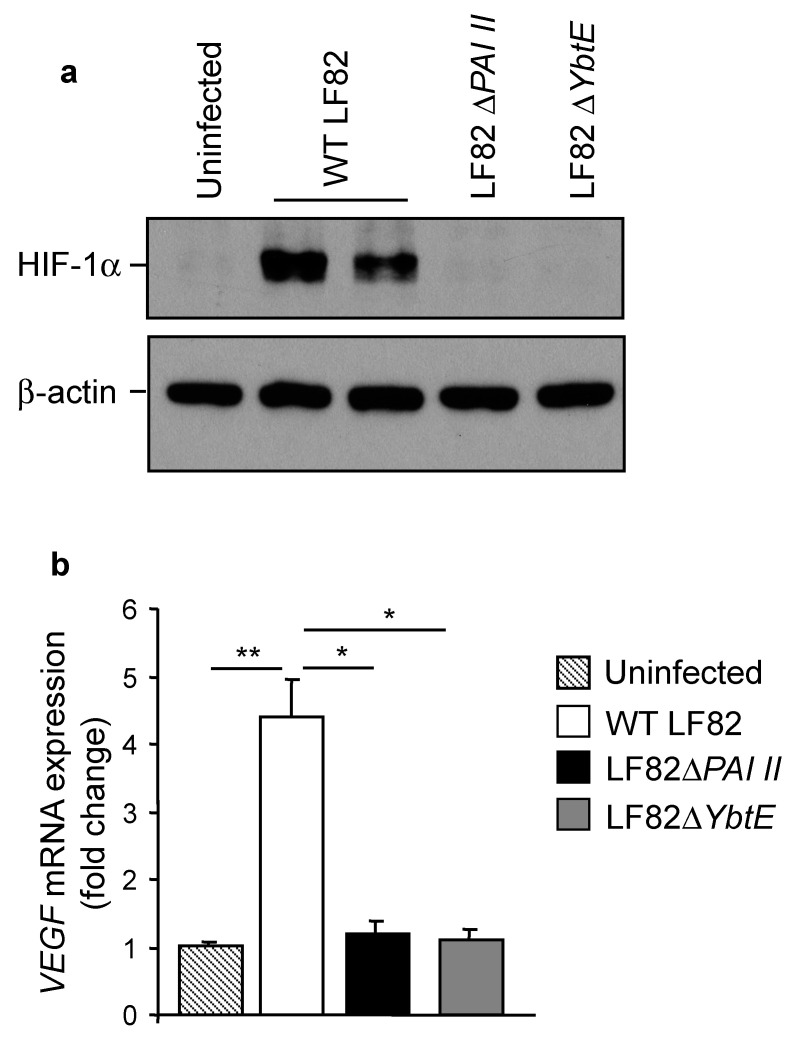
Yersiniabactin is required for AIEC LF82-induced HIF-1α expression and activation in IECs. (**a**,**b**) T84 cells were infected with the WT LF82 strain or with one of the mutants AIECΔ*PAI II* and AIECΔ*YbtE* for 3 h, washed, and incubated with gentamicin for 3 h. (**a**) HIF-1α expression was analyzed by Western blot. (**b**) *VEGF* mRNA expression level was assessed by qRT-PCR. Data are means ± SEM of six replicates and are representative of two independent experiments. * *p* < 0.05, ** *p* ≤ 0.01 by one-way Anova, followed by a post-test Bonferroni.

It was reported that during infection with human pathogenic bacteria, bacterial siderophores are implicated in hypoxia-independent activation of the transcription factor HIF-1α [43]. As shown in Figure 4a, infection with the wild-type LF82 strain induced HIF-1α expression in T84 cells, and this was not observed for infection with the mutants LF82Δ*PAI II* or LF82Δ*YbtE*. Furthermore, LF82 infection induced an increase in the mRNA expression level of *VEGF*, one of the well-known target of HIF-1α [44], but infection with LF82Δ*PAI II* or LF82Δ*YbtE* failed to do so (Figure 4b). These results suggested that yersiniabactin of AIEC is crucial for expression and activation of HIF-1α.

### 2.6. AIEC Induced HIF-1α Expression by Chelating Iron Rather Than by Inducing Hypoxia

**Figure 5:**
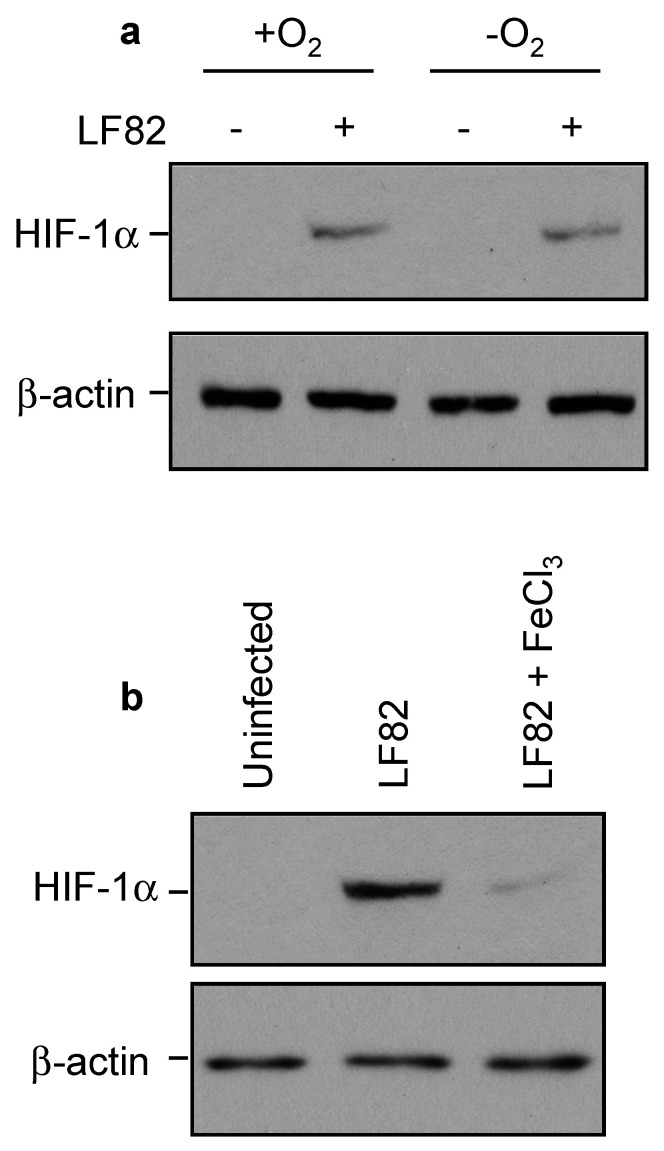
AIEC LF82 activates HIF-1α by chelating iron rather than by inducing hypoxia. (**a**) T84 cells were grown on regular plastic (−O_2_) or gas-permeable (+O_2_) dishes and then infected with the wild-type AIEC LF82 strain for 3 h, washed, and incubated with gentamicin for 3 h. HIF-1α expression was assessed by Western blot. (**b**) T84 cells were grown on regular plastic and then infected with the wild-type LF82 strain as in (**a**) in normal culture conditions or in the presence of 200 µM of FeCl_3_. HIF-1α expression was assessed by Western blot.

It is known that HIF-1α can be activated by either hypoxia or iron deprivation [45,46]. In order to elucidate the mechanism underlying LF82-induced HIF-1α expression, T84 cells were grown on regular plastic or gas-permeable dishes. We observed a strong expression of HIF-1α upon LF82 infection in both conventional and gas-permeable cell culture dishes (Figure 5a), indicating that hypoxia was not involved in LF82-induced HIF-1α expression. To assess whether iron deprivation was responsible for LF82-induced HIF-1α expression, T84 cells were infected with LF82 in the presence or absence of FeCl_3_. As shown in Figure 5b, the presence of iron strongly diminished LF82-induced HIF-1α expression. Together, these results indicated that AIEC LF82 activated HIF-1α expression by chelating iron.

### 2.7. HIF-1α Controls AIEC Intracellular Replication by Modulating Host Autophagy

**Figure 6:**
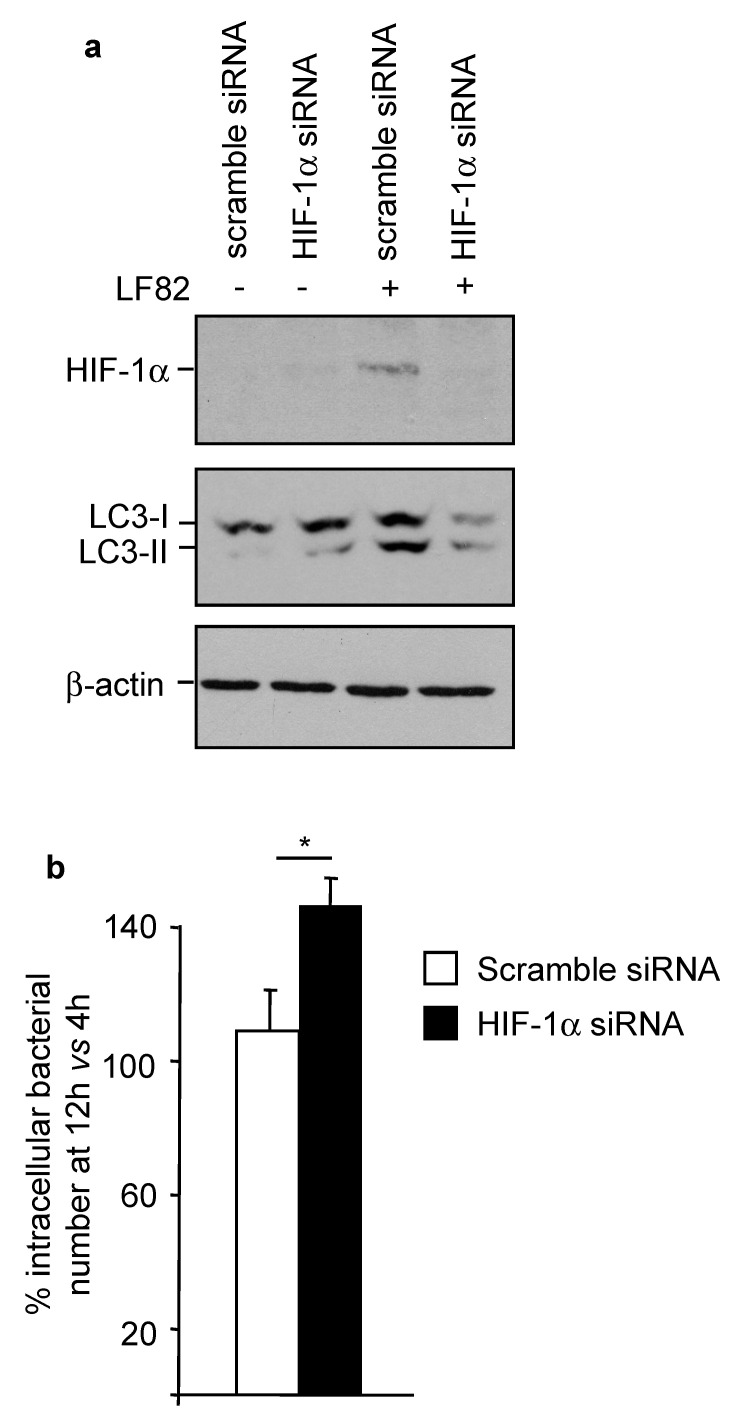
HIF-1α controls AIEC LF82 intracellular replication by modulating host autophagy. (**a**,**b**) T84 cells were transfected with 50 nM of *HIF-1α* siRNA or a scramble siRNA for 24 h and then infected or not with the wild-type AIEC LF82 strain for 3 h. Cells were washed and then incubated with gentamicin. (**a**) Expression levels of HIF-1α and LC3 were analyzed by Western blot at 6 h (3 h infection + 3 h with gentamicin) post-infection. (**b**) The number of intracellular LF82 was counted on LB agar plates and is presented as the ratio of intracellular bacteria at 12 h (3 h infection + 9 h in gentamicin) compared to 4 h (3 h infection + 1 h in gentamicin) post-infection. Data are means ± SEM of six replicates and are representative of two independent experiments. * *p* < 0.05 by Mann–Whitney test.

Finally, we sought to investigate the functional impact of AIEC-induced HIF-1α expression. To analyze the role of HIF-1α in AIEC-induced autophagy, before infection, T84 cells were transfected with an siRNA directed against *HIF-1α* mRNA or a scramble siRNA. As shown in Figure 6a, in cells transfected with the scramble siRNA, infection with LF82 induced HIF-1α expression and autophagy, as shown by the increased LC3-II level. *HIF-1α* siRNA inhibited LF82-induced HIF-1α expression and subsequently suppressed autophagy activation (Figure 6a). The decrease in autophagy activation in *HIF-1α*-siRNA-transfected cells was correlated with an increase in LF82 intracellular replication (Figure 6b). Together, our results suggest that HIF-1α controls AIEC LF82 intracellular replication by modulating host autophagy.

## 3. Discussion

In the present study, we observed that commensal *E. coli* could harbor the *Yersinia* HPI encoding yersiniabactin, confirming previously reported data [34]. Interestingly, we found that the yersiniabactin siderophore was over-represented in CD-associated AIEC compared to commensal *E. coli*. This result confirmed previous data showing increased prevalence of yersiniabactin iron transport genes in AIEC strains (presence in 6/8 strains) relative to non-pathogenic *E. coli* strains (presence in 1/12 strains) [40]. In the current study, our data, using 73 AIEC and 40 commensal *E. coli* strains, strongly support the overrepresentation of yersiniabactin siderophore-encoding genes among the AIEC strains, suggesting that this siderophore could be implicated in the pathogenic properties of the bacteria.

Given the overrepresentation of yersiniabactin among the AIEC strains and that CD patients suffer from chronic iron deficiency [47], we can speculate that yersiniabactin might confer the bacteria an advantage in the “iron competition” inside the intestinal tract. Indeed, we showed that the loss of yersiniabactin, while it did not affect the growth of AIEC LF82 in culture media, was a disadvantage for the bacteria when they were cultured with the wild-type LF82 strain having yersiniabactin. This supported the hypothesis that in a complex and hyper-competitive environment, such as the gastrointestinal tract, this siderophore could be crucial for AIEC colonization. This was confirmed by in vitro and in vivo experiments showing that the loss of yersiniabactin led to a decrease in the number of AIEC that adhered to and invaded IECs and to the reduction of AIEC colonization in the gut of CEABAC10 transgenic mice. This is in accordance with previous data showing that in *Enterobacteriaceae*, HPIs contribute to fitness and virulence of the bacteria in vivo [43,48,49]. Recently, the deleterious effect of yersiniabactin of the mouse AIEC strain NC101 was demonstrated [50]. Indeed, in germ-free *il10^−/−^* mice, this siderophore promoted a profibrogenic host response, suggesting a direct link between intestinal *E. coli* and the induction of inflammation-associated fibrosis [50].

*Y. enterocolitica* has been shown to activate the transcriptional factor HIF-1α in vivo [43]. This activation is protective, since *hif1a^−/−^* mice are more susceptible to *Y. enterocolitica* infection than wild-type mice. Furthermore, yesrsiniabactin derived from *Y. enterocolitica* activates HIF-1α in vitro [43]. In our study, we also observed an intestinal epithelial response to yersiniabactin leading to HIF-1α activation. We observed that the presence of this siderophore in AIEC activated HIF-1α, leading to autophagy activation and consequently to a better control of AIEC intracellular replication. We have previously shown that autophagy plays a crucial role in the interaction between AIEC and the host. Upon AIEC infection, autophagy is induced in host cells via the activation of the metabolic stress response pathway EIF2AK4–EIF2A–ATF4 [24]. However, AIEC can subvert autophagy by up-regulating the levels of specific microRNAs that target autophagy and inhibit this process [26]. AIEC also inhibit autophagy by impairing host SUMOylation, a eukaryotic-reversible post-translational modification [27]. These mechanisms consequently lead to increases in AIEC intracellular replication and AIEC-induced inflammation in vitro and in vivo [24,25,26,27]. However, in our previous reports, the bacterial virulence factor responsible for these effects was not explored. Here, we demonstrated that a functional siderophore yersiniabactin is implicated in the activation of autophagy upon infection with AIEC. Thus, a pathogenicity island could be, at some point, a disadvantage for the bacteria, since its presence may “alarm” the host defense mechanisms, such as autophagy.

**Figure 7:**
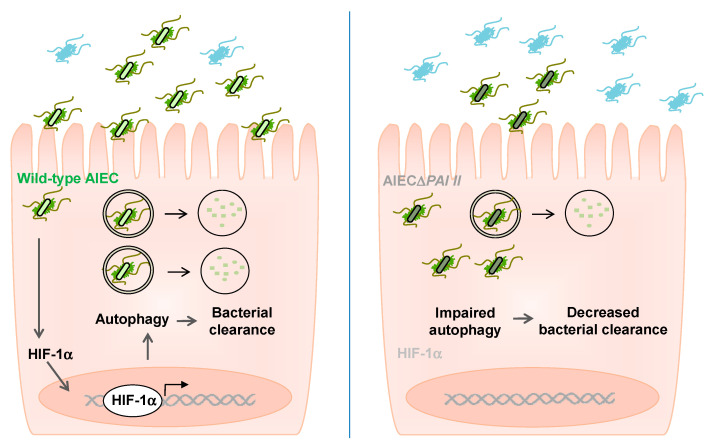
Hypothetical model for the involvement of PAI II in the interaction of AIEC with intestinal epithelial cells. Left panel: PAI II confers AIEC a high ability to compete with other bacteria for growth and adhesion to IECs. AIEC PAI II-encoded yersiniabactin activates autophagy via a HIF-1α-dependent mechanism, leading to bacterial clearance. Right panel: AIEC invalidated for PAI II (AIECΔ*PAI II*) have a lower ability to compete with other bacteria for adhesion to intestinal epithelial cells. Autophagy in IECs infected with AIECΔ*PAI II* is reduced, leading to impaired bacterial clearance, compared to that in IECs infected with wild-type AIEC. Thus, the presence of yersiniabactin, which is an advantage for AIEC to grow in a competitive environment, could be a disadvantage for the bacteria, as it activates autophagy-mediated bacterial clearance.

In conclusion, our study highlights the complex crosstalk between AIEC and the host and the dual effect of PAI II-encoded yersiniabactin. We showed that yersiniabactin, which confers the advantage to AIEC to grow in a complex environment and gain access to competitive ecological niches, could be, at some point, a disadvantage when it activates signaling pathways leading to bacterial clearance, such as autophagy (Figure 7).

## 4. Materials and Methods

### 4.1. Bacterial Strains

The AIEC reference strain LF82 was isolated from the ileal mucosa of a CD patient [7]. Bacteria were grown in Luria-Bertani (LB) broth or on LB agar plates overnight at 37 °C.

### 4.2. Screening for the Presence of PAI II by PCR

Bacteria were grown in LB overnight, then boiled, and PCR was performed using GoTaq DNA Polymerase (Promega, Charbonnières-les-Bains, France) following the manufacturer’s instructions. Two different sets of primers, each amplifying a different PAI II gene, were used.

- LF82_p298_Forward_: 5′-GCGCCCAGACGAATGTTATT-3′;
- LF82_p298_Reverse_: 5′-CAACACCTCTCATTACGCGG-3′;
- LF82_p306_Forward_: 5′-TTCAGGCAGTTTGTGGGAGA-3′;
- LF82_p306_Reverse_: 5′-TCACTCTTACCGCCAACCAT-3′.

### 4.3. Construction of AIEC LF82 Chromosomal Mutant Invalidated for PAI I

The entire PAI II was deleted in the AIEC LF82 strain by homologous recombination, as previously described [51,52]. Briefly, 540 bp of the LF82_p294 gene (the first gene of the PAI II) and 607 bp of the LF82_p308 gene (the last gene of the PAI II) were amplified by PCR using the following primers:

- LF82_p294_Forward_: 5′-GGGCCCGAATTCGCTAAGTAATATGCGCCCCG-3′; LF82_p294_Reverse_: 5′-CGATGTTTCGGAGTCAGTCGCGGATACCTTCACGTTGCTG-3′;
- LF82_p308_Forward_: 5′-CAGCAACGTGAAGGTATCCGCGACTGACTCCGAAACATCG-3′;
- LF82_p308_Reverse_: 5′-GGGCCCGGATCCAACGAATTTCCAGGTCGTCG-3′.

The two PCR fragments were assembled by PCR and cloned into the pST76 plasmid using BamHI and EcoRI restriction sites. This vector possesses a thermosensible Ori and an I-SceI restriction site. By increasing the temperature, we forced pST76 insertion inside the AIEC LF82 chromosome. This insertion involved a homologous recombination between PAI II and sequences cloned inside the vector. Bacteria were then transformed by an I-SceI enzyme-producing pDAI vector. Cleavage of the I-SceI site inside pST76 can induced homologous recombination, resulting in the loss of PAI II (AIECΔ*PAI II*). Deletion of PAI II was verified by PCR using the primers located inside the island:

- LF82_p298_Forward_: 5′-GCGCCCAGACGAATGTTATT-3′;
- LF82_p298_Reverse_: 5′-CAACACCTCTCATTACGCGG-3′;
- LF82_p306_Forward_: 5′-TTCAGGCAGTTTGTGGGAGA-3′;
- LF82_p306_Reverse_: 5′-TCACTCTTACCGCCAACCAT-3′.

For *YbtE* gene deletion, we used the method described by Datsenko et al. [53] and modified by Chaveroche et al. [54], comprising the replacement of the *YbtE* gene by a selective antibiotic cassette (kanamycin) generated by PCR using the following primers:

- YbtEKana_Forward_: 5′-ATGTGCATCCCGCTGTGGCCCGCCCGGAACGGCAATACTGCGCATCTGGGTAGGCTGGAGCTGCTTCG-3′;
- YbtEKana_Reverse_: 5′-TCACCTTTCTGAAGTACTGGGCTGTTTTTGCCACGCGGTCATTGCAGCATATGAATATCCTCCTTAGTTC-3′.

Briefly, LF82 was transformed with pKOBEG, a plasmid encoding the Red proteins that protect linear DNA from degradation in bacteria. The plasmid was maintained in bacteria at 30 °C with 25 µg/mL of chloramphenicol, and Red protein expression was induced using 1 mM of L-arabinose (Sigma-Aldrich, Saint-Quentin Fallavier, France). Then, the YbtEKana PCR product was electroporated in previously refrigerated glycerol-washed LF82. The resulting LF82Δ*YbtE* isogenic mutant (Km^R^) was selected on LB containing 50 µg/mL of kanamycin. The replacement of the *YbtE* gene by the kanamycin resistance cassette was confirmed by PCR.

### 4.4. Cell Culture

The intestinal epithelial cell line T84 (CCL-248; ATCC, Molsheim, France) was maintained in an atmosphere containing 5% CO_2_ at 37 °C in the culture medium recommended by ATCC (Molsheim, France). T84 cells were cultivated using conventional polystyrene dishes or special gas-permeable dishes with a hydrophilic tissue culture-treated bottom membrane (Lumox; Sarstedt, Marnay, France) as previously used [43].

### 4.5. Infection and Gentamicin Protection Assay

T84 cells were seeded on 12- or 24-well plates and infected at a multiplicity of infection (MOI) of 10 bacteria per cell. Gentamicin protection assay was performed as previously described [26]. Briefly, after 3 h of incubation with the bacteria in the culture medium without antibiotics, the cells were washed with PBS and incubated with the culture medium containing 100 μg/mL of gentamicin for 1 h, 3 h, or 9 h. When indicated, the cells were pre-treated with Bafilomycin A1 (Sigma-Aldrich, Saint-Quentin Fallavier, France) at 50 nM for 30 min before infection, and then Bafilomycin A1 was maintained in the medium during infection. The cells were lysed with 1% Triton X-100 (Sigma-Aldrich, Saint-Quentin Fallavier, France) in deionized water. Samples were serially diluted and plated onto LB agar plates, and the number of bacteria was determined by counting the colony-forming units (CFU).

In the competitive experiment, T84 cells were infected with 10 wild-type LF82 bacteria and 10 mutant bacteria (LF82*ΔPAI II* or LF82Δ*YbtE*) per cell as described above.

### 4.6. Protein Extraction and Western Blot Analysis

Cells were lysed in radioimmune precipitation assay buffer (150 mM NaCl, 0.5% sodium deoxycholate, 50 mM Tris-HCl, pH 8, 0.1% SDS, 0.1% Nonidet P-40) supplemented with protease inhibitors (Roche, Boulogne-Billancourt, France). Proteins were separated on SDS/PAGE gels, transferred to nitrocellulose membranes (Amersham Biosciences, Velizy-Villacoublay, France), and blocked with 5% non-fat milk in PBS containing 0.1% Tween-20. Membranes were then incubated overnight at 4 °C with the relevant primary antibodies: anti-HIF-1α (Thermo Scientific, Illkirch, France), anti-LC3 (Sigma-Aldrich, Saint-Quentin Fallavier, France), anti-p62 (Cell Signaling Technology, Saint-Cyr-L’École, France), and anti-β-actin (Cell Signaling Technology, Saint-Cyr-L’École, France). After washes, membranes were incubated with the appropriate HRP-conjugated secondary antibodies (Cell Signaling Technology, Saint-Cyr-L’École, France). Blots were detected using the Enhanced Chemiluminescence Detection kit (Amersham Biosciences, Velizy-Villacoublay, France), revealed using the ChemiDocTM XRS System (Bio-Rad, Marnes-la-Coquette, France), and quantified using the Image Lab Software (Bio-Rad, Marnes-la-Coquette, France).

### 4.7. Quantitative Real-Time RT-PCR (qRT-PCR)

Total RNAs were isolated using the TRIzol reagent (Invitrogen, Illkirch, France) following the manufacturer’s instruction. In this experiment, 2 µg of total RNA were reversely transcribed using the first-strand cDNA synthesis kit (Euromedex, Souffelweyersheim, France) to quantify mRNA expression levels. Quantitative (q)RT-PCR was performed using SYBR Green Master Mix (Euromedex, Souffelweyersheim, France) on a Mastercycler Realplex^4^ (Eppendorf, Montesson, France) using the following primers:

- *VEGF*_Forward_: 5′-TGGAGCGTGTACGTTGGTG-3′;
- *VEGF*_Reverse_: 5′-GCGAGTCTGTGTTTTTGCAG-3′;
- *RPLP0*_Forward_: 5′- TCTGCATTCTCGCTTCCTGG-3′;
- *RPLP0*_Reverse_: 5′- CAGGACTCGTTTGTACCCGT-3′.

*RPLP0* was used as a house-keeping gene. Fold-induction was calculated using the comparative threshold cycle number (*Ct*) method as follows: ∆∆*Ct* = (*Ct_VEGF_* − *Ct_RPLP0_*)_test condition_ − (*Ct_VEGF_* − *Ct_RPLP0_*)_control condition_, and the final data were derived from 2^−∆∆*Ct*^.

### 4.8. Transfection Experiments

Cells cultured on 12-well plates were transfected with 50 nM of HIF-1α siRNA (5′-GGGUAAAGAACAAAACACAtt-3′, Ambion, Illkirch, France) or scramble siRNA (Ambion, Illkirch, France) using Lipofectamine 2000 (Invitrogen) and OPTI-MEM serum-reduced medium (Invitrogen, Illkirch, France) according to the manufacturer’s instructions. Cells were changed to fresh culture medium after 8 h of transfection and infected 24 h later.

### 4.9. Transmission Electron Microscopy

Bacteria were grown overnight at 37 °C in LB without shaking, placed for 1 min on carbon-Formvar copper grids (Electron Microscopy Sciences, Hatfield, PA, USA), and negatively stained for 1 min with phosphotungstic acid pH 6.0. Grids were examined with a Hitachi H-7650 transmission electron microscope (Hitachi, Krefeld, Germany).

### 4.10. Competition in Vitro

Bacteria (LF82, LF82Δ*PAI II* and LF82Δ*YbtE*) were grown overnight in LB at 37 °C. In total, 100 bacteria of wild-type LF82 and the mutant were mixed in 10 mL of LB and then incubated at 37 °C. At the indicated time, serial dilutions were spread on LB agar plates with or without antibiotics (to distinguish the different bacterial strains). All the inocula were spread to verify the quantity of bacteria mixed.

### 4.11. Competition In Vivo

Experiments were performed using 6-week-old CEABAC10 transgenic male mice housed in the specific pathogen-free animal facility at the University Clermont Auvergne. Mice were fed standard chow ad libitum throughout the experiments, had free access to sterile water, and were subjected to 12–12 light/dark cycles. They received oral gavage of a 200 µL suspension containing the same amounts of wild-type LF82 and LF82Δ*PAI II* (10^9^ CFU each). Feces and tissues were collected at the indicated times and crushed, and serial dilutions were plated either on LB agar plates containing ampicillin (100 µg/mL) and erythromycin (25 µg/mL) to detect LF82 or on LB agar plates containing ampicillin (100 µg/mL), erythromycin (25 µg/mL), and rifampicin (100 µg/mL) to detect AIECΔ*PAI II*.

### 4.12. Statistical Analysis

Values were expressed as means ± SEM. Statistical analysis between two groups was performed with GraphPad Prism version 5.01 software (San Diego, CA, USA) using a two-tailed Student’s *t*-test analysis or a non-parametric Mann–Whitney test depending on D’Agostino–Pearson omnibus normality test. Statistical analysis between more than two groups was performed using one-way ANOVA, followed by a Bonferroni post-test. To analyze the prevalence of PAI II between AIEC strains and commensal *E. coli* strains, a chi-square test was used. A *p* value less than 0.05 was considered statistically significant.

## Data Availability

The data presented in this study are available on request from the corresponding author.

## References

[1] Palmela C., Chevarin C., Xu Z., Torres J., Sevrin G., Hirten R., Barnich N., Ng S.C., Colombel J.-F. (2018). Adherent-invasive escherichia coli in inflammatory bowel disease. Gut.

[2] Carrière J., Darfeuille-Michaud A., Nguyen H.T.T. (2014). Infectious etiopathogenesis of Crohn’s disease. World J. Gastroenterol..

[3] Darfeuille-Michaud A., Boudeau J., Bulois P., Neut C., Glasser A.-L., Barnich N., Bringer M.A., Swidsinski A., Beaugerie L., Colombel J.-F. (2004). High prevalence of adherent-invasive escherichia coli associated with ileal mucosa in Crohn’s disease. Gastroenterology.

[4] O’Brien C.L., Bringer M.A., Holt K.E., Gordon D.M., Dubois A.L., Barnich N., Darfeuille-Michaud A., Pavli P. (2017). Comparative genomics of Crohn’s disease-associated adherent-invasive Escherichia coli. Gut.

[5] Martinez-Medina M., Aldeguer X., Lopez-Siles M., González-Huix F., López-Oliu C., Dahbi G., Blanco J.E., Blanco J., Garcia-Gil L.J., Darfeuille-Michaud A. (2009). Molecular Diversity of Escherichia coli in the human gut: New ecological evidence supporting the role of adherent-invasive *E. coli* (AIEC) in Crohn’s disease. Inflamm. Bowel Dis..

[6] Martin H.M., Campbell B.J., Hart C.A., Mpofu C., Nayar M., Singh R., Englyst H., Williams H.F., Rhodes J.M. (2004). Enhanced Escherichia coli adherence and invasion in Crohn’s disease and colon cancer. Gastroenterology.

[7] Darfeuille-Michaud A., Neut C., Barnich N., Lederman E., Di Martino P., Desreumaux P., Gambiez L., Joly B., Cortot A., Colombel J.F. (1998). Presence of adherent Escherichia coli strains in ileal mucosa of patients with Crohn’s disease. Gastroenterology.

[8] Boudeau J., Glasser A.L., Masseret E., Joly B., Darfeuille-Michaud A. (1999). Invasive ability of an Escherichia coli strain isolated from the ileal mucosa of a patient with Crohn’s disease. Infect. Immun..

[9] Glasser A.L., Boudeau J., Barnich N., Perruchot M.H., Colombel J.F., Darfeuille-Michaud A. (2001). Adherent invasive Escherichia coli strains from patients with Crohn’s disease survive and replicate within macrophages without inducing host cell death. Infect. Immun..

[10] Chervy M., Barnich N., Denizot J. (2020). Adherent-invasive *E. coli*: Update on the lifestyle of a troublemaker in Crohn’s disease. Int. J. Mol. Sci..

[11] Delmas J., Gibold L., Faïs T., Batista S., Leremboure M., Sinel C., Vazeille E., Cattoir V., Buisson A., Barnich N. (2019). Metabolic Adaptation of adherent-invasive Escherichia coli to exposure to bile salts. Sci. Rep..

[12] Gibold L., Garenaux E., Dalmasso G., Gallucci C., Cia D., Mottet-Auselo B., Faïs T., Darfeuille-Michaud A., Nguyen H.T.T., Barnich N. (2015). The vat-AIEC protease promotes crossing of the intestinal mucus layer by Crohn’s disease-associated Escherichia coli. Cell. Microbiol..

[13] Sevrin G., Massier S., Chassaing B., Agus A., Delmas J., Denizot J., Billard E., Barnich N. (2020). Adaptation of adherent-invasive *E. coli* to gut environment: Impact on flagellum expression and bacterial colonization ability. Gut Microbes.

[14] Low D., Tran H.T., Lee I.A., Dreux N., Kamba A., Reinecker H.C., Darfeuille-Michaud A., Barnich N., Mizoguchi E. (2013). Chitin-binding domains of Escherichia coli chia mediate interactions with intestinal epithelial cells in mice with colitis. Gastroenterology.

[15] Boudeau J., Barnich N., Darfeuille-Michaud A. (2001). Type 1 pili-mediated adherence of Escherichia coli strain lf82 isolated from Crohn’s disease is involved in bacterial invasion of intestinal epithelial cells. Mol. Microbiol..

[16] Dreux N., Denizot J., Martinez-Medina M., Mellmann A., Billig M., Kisiela D., Chattopadhyay S., Sokurenko E., Neut C., Gower-Rousseau C. (2013). Point mutations in FimH adhesin of Crohn’s disease-associated adherent-invasive Escherichia coli enhance intestinal inflammatory response. PLoS Pathog.

[17] Barnich N., Carvalho F.A., Glasser A.L., Darcha C., Jantscheff P., Allez M., Peeters H., Bommelaer G., Desreumaux P., Colombel J.F. (2007). CEACAM6 acts as a receptor for adherent-invasive *E. coli*, supporting ileal mucosa colonization in Crohn disease. J. Clin. Investig..

[18] Carvalho F.A., Barnich N., Sivignon A., Darcha C., Chan C.H.F., Stanners C.P., Darfeuille-Michaud A. (2009). Crohn’s disease adherent-invasive Escherichia coli colonize and induce strong gut inflammation in transgenic mice expressing human CEACAM. J. Exp. Med..

[19] Nguyen H.T.T., Lapaquette P., Bringer M.A., Darfeuille-Michaud A. (2013). Autophagy and Crohn’s disease. J. Innate Immun..

[20] Larabi A., Barnich N., Nguyen H.T.T. (2020). New insights into the interplay between autophagy, gut microbiota and inflammatory responses in IBD. Autophagy.

[21] Lapaquette P., Glasser A.L., Huett A., Xavier R.J., Darfeuille-Michaud A. (2010). Crohn’s disease-associated adherent-invasive *E. coli* are selectively favoured by impaired autophagy to replicate intracellularly. Cell. Microbiol..

[22] Lapaquette P., Bringer M.A., Darfeuille-Michaud A. (2012). Defects in autophagy favour adherent-invasive Escherichia coli persistence within macrophages leading to increased pro-inflammatory response. Cell. Microbiol..

[23] Brest P., Lapaquette P., Souidi M., Lebrigand K., Cesaro A., Vouret-Craviari V., Mari B., Barbry P., Mosnier J.F., Hébuterne X. (2011). A synonymous variant in IRGM alters a binding site for MiR-196 and causes deregulation of IRGM-dependent xenophagy in Crohn’s disease. Nat. Genet..

[24] Bretin A., Carrière J., Dalmasso G., Bergougnoux A., B’chir W., Maurin A.C., Müller S., Seibold F., Barnich N., Bruhat A. (2016). Activation of the EIF2AK4-EIF2A/EIF2α-ATF4 pathway triggers autophagy response to Crohn disease-associated adherent-invasive Escherichia coli infection. Autophagy.

[25] Bretin A., Lucas C., Larabi A., Dalmasso G., Billard E., Barnich N., Bonnet R., Nguyen H.T.T. (2018). AIEC infection triggers modification of gut microbiota composition in genetically predisposed mice, contributing to intestinal inflammation. Sci. Rep..

[26] Nguyen H.T.T., Dalmasso G., Müller S., Carrière J., Seibold F., Darfeuille-Michaud A. (2014). Crohn’s disease-associated adherent invasive Escherichia coli modulate levels of micrornas in intestinal epithelial cells to reduce autophagy. Gastroenterology.

[27] Dalmasso G., Nguyen H.T.T., Faïs T., Massier S., Barnich N., Delmas J., Bonnet R. (2019). Crohn’s disease-associated adherent-invasive Escherichia coli manipulate host autophagy by impairing SUMOylation. Cells.

[28] Carrière J., Bretin A., Darfeuille-Michaud A., Barnich N., Nguyen H.T.T. (2016). Exosomes released from cells infected with Crohn’s disease-associated adherent-invasive Escherichia coli activate host innate immune responses and enhance bacterial intracellular replication. Inflamm. Bowel Dis..

[29] Larabi A., Dalmasso G., Delmas J., Barnich N., Nguyen H.T.T. (2020). Exosomes transfer MiRNAs from cell-to-cell to inhibit autophagy during infection with Crohn’s disease-associated adherent-invasive *E. coli*. Gut Microbes.

[30] Evstatiev R., Gasche C. (2012). Iron sensing and signalling. Gut.

[31] Wilson B.R., Bogdan A.R., Miyazawa M., Hashimoto K., Tsuji Y. (2016). Siderophores in iron metabolism: From mechanism to therapy potential. Trends Mol. Med..

[32] Ellermann M., Arthur J.C. (2017). Siderophore-mediated iron acquisition and modulation of host-bacterial interactions. Free Radic. Biol. Med..

[33] Khan A., Singh P., Srivastava A. (2017). Synthesis, Nature and utility of universal iron chelator-siderophore: A review. Microbiol. Res..

[34] Schubert S., Rakin A., Heesemann J. (2004). The yersinia high-pathogenicity island (HPI): Evolutionary and functional aspects. Int. J. Med. Microbiol..

[35] Miquel S., Peyretaillade E., Claret L., de Vallée A., Dossat C., Vacherie B., Zineb E.H., Segurens B., Barbe V., Sauvanet P. (2010). Complete genome sequence of Crohn’s disease-associated adherent-invasive *E. coli* strain LF82. PLoS ONE.

[36] Wright E.K., Kamm M.A., Teo S.M., Inouye M., Wagner J., Kirkwood C.D. (2015). Recent advances in characterizing the gastrointestinal microbiome in Crohn’s disease: A systematic review. Inflamm. Bowel Dis..

[37] Faber F., Bäumler A.J. (2014). The impact of intestinal inflammation on the nutritional environment of the gut microbiota. Immunol. Lett..

[38] Céspedes S., Saitz W., Del Canto F., De la Fuente M., Quera R., Hermoso M., Muñoz R., Ginard D., Khorrami S., Girón J. (2017). Genetic diversity and virulence determinants of Escherichia coli strains isolated from patients with Crohn’s disease in Spain and Chile. Front. Microbiol..

[39] Rakitina D.V., Manolov A.I., Kanygina A.V., Garushyants S.K., Baikova J.P., Alexeev D.G., Ladygina V.G., Kostryukova E.S., Larin A.K., Semashko T.A. (2017). Genome analysis of *E. coli* isolated from Crohn’s disease patients. BMC Genomics.

[40] Dogan B., Suzuki H., Herlekar D., Sartor R.B., Campbell B.J., Roberts C.L., Stewart K., Scherl E.J., Araz Y., Bitar P.P. (2014). Inflammation-associated adherent-invasive Escherichia coli are enriched in pathways for use of propanediol and iron and M-cell translocation. Inflamm. Bowel Dis..

[41] Bearden S.W., Fetherston J.D., Perry R.D. (1997). Genetic organization of the yersiniabactin biosynthetic region and construction of avirulent mutants in yersinia pestis. Infect. Immun..

[42] Klionsky D.J., Abdel-Aziz A.K., Abdelfatah S., Abdellatif M., Abdoli A., Abel S., Abeliovich H., Abildgaard M.H., Abudu Y.P., Acevedo-Arozena A. (2021). Guidelines for the use and interpretation of assays for monitoring autophagy. Autophagy.

[43] Hartmann H., Eltzschig H.K., Wurz H., Hantke K., Rakin A., Yazdi A.S., Matteoli G., Bohn E., Autenrieth I.B., Karhausen J. (2008). Hypoxia-independent activation of HIF-1 by enterobacteriaceae and their siderophores. Gastroenterology.

[44] Forsythe J.A., Jiang B.H., Iyer N.V., Agani F., Leung S.W., Koos R.D., Semenza G.L. (1996). Activation of Vascular endothelial growth factor gene transcription by hypoxia-inducible factor 1. Mol. Cell. Biol..

[45] Pugh C.W., Ratcliffe P.J. (2003). Regulation of angiogenesis by hypoxia: Role of the HIF system. Nat. Med..

[46] Semenza G.L. (2001). HIF-1, O2, and the 3 PHDs: How animal cells signal hypoxia to the nucleus. Cell.

[47] Stein J., Hartmann F., Dignass A.U. (2010). Diagnosis and management of iron deficiency anemia in patients with IBD. Nat. Rev. Gastroenterol. Hepatol..

[48] Bachman M.A., Oyler J.E., Burns S.H., Caza M., Lépine F., Dozois C.M., Weiser J.N. (2011). Klebsiella pneumoniae yersiniabactin promotes respiratory tract infection through evasion of lipocalin 2. Infect. Immun..

[49] Heesemann J., Hantke K., Vocke T., Saken E., Rakin A., Stojiljkovic I., Berner R. (1993). Virulence of Yersinia enterocolitica is closely associated with siderophore production, expression of an iron-repressible outer membrane polypeptide of 65,000 Da and pesticin sensitivity. Mol. Microbiol..

[50] Ellermann M., Gharaibeh R.Z., Fulbright L., Dogan B., Moore L.N., Broberg C.A., Lopez L.R., Rothemich A.M., Herzog J.W., Rogala A. (2019). Yersiniabactin-Producing adherent/invasive Escherichia coli promotes inflammation-associated fibrosis in gnotobiotic Il10-/- mice. Infect. Immun..

[51] Massier S., Miquel S., Dreux N., Agus A., Bouhours S., Denizot J., Darfeuille-Michaud A., Barnich N. (2015). Involvement of type VI secretion systems in virulence of adherent-invasive Escherichia coli isolated from patients with Crohn’s disease. J. Crohns Colitis.

[52] Pósfai G., Kolisnychenko V., Bereczki Z., Blattner F.R. (1999). Markerless gene replacement in Escherichia coli stimulated by a double-strand break in the chromosome. Nucleic Acids Res..

[53] Datsenko K.A., Wanner B.L. (2000). One-step inactivation of chromosomal genes in Escherichia coli K-12 using PCR products. Proc. Natl. Acad. Sci. USA.

[54] Chaveroche M.K., Ghigo J.M., d’Enfert C. (2000). A Rapid method for efficient gene replacement in the filamentous fungus aspergillus nidulans. Nucleic Acids Res..

